# Solvent Interaction Analysis: A New Lens for Protein Structure and Diagnostics

**DOI:** 10.3390/ijms27156645

**Published:** 2026-07-25

**Authors:** Boris Y. Zaslavsky, Mark Stovsky, Vladimir N. Uversky

**Affiliations:** 1 Cleveland Diagnostics, 3615 Superior Ave., Cleveland, OH 44114, USA; mark.stovsky@clevelanddx.com; 2 Department of Molecular Medicine and Byrd Alzheimer’s Research Institute, Morsani College of Medicine, University of South Florida, 12901 Bruce B. Downs Blvd., Tampa, FL 33612, USA

**Keywords:** aqueous two-phase systems, solvent interaction analysis, biomarker, phase separation, solvent properties, protein properties, proteoforms, prostate-specific antigen

## Abstract

Aqueous two-phase systems (ATPSs) provide a versatile, fully aqueous platform for probing solute–water interactions and protein structure. This review first surveys the diversity and phase behavior of biphasic aqueous systems formed by polymers and salts. We describe how phase diagrams characterize ATPS formation and composition and how both polymer chemistry and salt identity, rather than molecular size alone, govern phase separation by modulating the solvent properties of water. Building on a modified binodal model, we show that phase separation and solute partitioning can be understood in terms of changes in aqueous solvent dipolarity/polarizability, hydrogen-bond donor/acceptor properties, hydrophobicity, and electrostatics, quantified via solvatochromic probes and homologous solute series. These measurements underpin solvent interaction analysis (SIA), in which the partition coefficients of small molecules and proteins across panels of ATPSs are used to generate “structural signatures” that sensitively report on amino acid substitutions, conformational changes, aggregation, ligand binding, osmolyte effects, and post-translational modifications, independent of protein size. We discuss how SIA can be implemented in vial-, plate-, and microfluidic formats and combined with diverse analytical readouts (HPLC, MS, colorimetric assays, and immunoassays), and we contrast this structure-focused approach with conventional concentration-only proteomic and biomarker strategies. Particular emphasis is placed on structure-based biomarker discovery, where disease-relevant shifts in proteoform distributions—especially glycosylation changes—are often more informative than bulk protein levels and where SIA can complement or simplify complex glycomics and top-down proteomics workflows. As a case study, we describe the recently FDA-approved IsoPSA assay, which applies SIA principles to prostate-specific antigen by measuring cancer-associated structural alterations in circulating PSA via its partition behavior in a proprietary ATPS. IsoPSA generates a single index that discriminates between high-grade prostate cancer and benign and low-grade conditions. Prospective, longitudinal, and MRI-integrated clinical studies demonstrate that IsoPSA improves pre-biopsy risk stratification, reduces unnecessary biopsies, and provides robust negative and positive predictive values within the PSA “gray zone.” Collectively, the data support aqueous solvent interaction analysis as a broadly applicable, mechanistically grounded technology for protein characterization, drug–protein interaction studies, and structure-centric biomarker development, exemplified by the clinical translation of IsoPSA.

## 1. Introduction

Solvent interaction analysis (SIA) is an advanced proteomic technique that evaluates protein structures using aqueous two-phase systems (ATPSs). The SIA technique isolates different protein isoforms (proteoforms) using a set of customized biphasic solutions created by mixing specific polymers and salts in water. By measuring how a protein partitions between the two immiscible aqueous phases, SIA generates a unique structural signature that is highly sensitive to amino acid variations and independent of overall protein concentration. SIA evaluates protein structure, structural dynamics, conformational stability, and post-translational modifications (such as glycosylation and phosphorylation). As a result, this protein profiling method investigates biomarkers at the structural level rather than just measuring their overall concentration. This review describes recent developments in the SIA technique and its applications. We briefly describe different types of aqueous multiphase and two-phase systems, phase diagrams, the fundamentals of the physical chemistry of solute partitioning, the characterization of individual proteins using the SIA technique, the advantages of structure-based proteomic analysis of biomarkers, and the first SIA-based FDA-approved test for the clinical early detection of prostate cancer, thereby suggesting a paradigm change in clinical diagnostics. The clinical success of the SIA-based IsoPSA assay indicates a broader structural paradigm shift in clinical diagnostics. By treating protein structure, structural flexibility, conformation, post-translational modifications, and interactions as the primary biomarkers, SIA broadens the diagnostic window for complex diseases, in which structural abnormalities precede alterations in protein expression levels.

## 2. Types of Biphasic and Multiphasic Systems in Water

The formation of multiple aqueous phases when different water-soluble compounds are mixed represents a widespread phenomenon. The number and characteristics of the phases that are formed depend on the physicochemical properties of the constituent compounds. The complexity of these systems can be remarkable—Albertsson documented an aqueous system containing six polymers (including dextran sulfate, dextran, and four hydroxypropyl dextrans with varying substitution levels) that separated into 18 distinct phases [1]. A comprehensive catalog by Mace et al. identified over 300 phase-separated systems, ranging from two to six aqueous phases, generated from mixtures of various polymers and surfactants [2]. While the relationship between the number of phase-forming components and the resulting number of phases remains unclear, all these systems share an important characteristic: they are reversible with respect to component concentrations. Diluting such a system below critical thresholds causes the originally separated phases to merge into a single phase, whereas restoring the component concentrations regenerates the multi-phase system.

These systems have proven valuable for creating stable density gradients in aqueous media, with phase density differences as small as 0.001 g/cm^3^. Such gradients have enabled the separation of nanoparticles [3] and the diagnosis of medical conditions through erythrocyte fractionation, including sickle cell disease [4] and iron-deficiency anemia [4,5]. They have also facilitated the isolation of reticulocyte-enriched fractions from blood samples [6].

While multi-phase systems demonstrate the versatility of aqueous phase separation, most research has focused on simpler aqueous two-phase systems (ATPSs) formed by just two phase-forming compounds. In these binary systems, two immiscible aqueous phases emerge once the concentrations of both components exceed specific threshold values [1]. Under controlled environmental conditions (constant temperature and composition), ATPS formation occurs spontaneously and reversibly, producing thermodynamically stable equilibrium systems. A clear interface separates the two phases, each enriched in one of the phase-forming components, while both retain substantial water content (typically exceeding 80% on a molal basis) [1,7,8,9,10]. The kinetics of phase separation vary considerably with system composition: polymer–salt systems generally separate rapidly (within minutes), whereas polymer–polymer systems (such as PEG–Ficoll) typically require longer equilibration times (hours) [7,8,9,10].

ATPSs can also be formed in solutions of single compounds under appropriate conditions. Examples include thermo-responsive polymers [11,12], stimulus-responsive polypeptides [13,14], and surfactants [15,16,17]. In these cases, once a critical temperature threshold is reached, the system separates into two phases: one enriched in the compound and another essentially free of it.

The diversity of ATPS compositions is substantial. Common configurations include two different polymers [1,7,8,9,10], a polymer with a salt [1,7,8,9,10] or surfactant [18,19], two different surfactants [20], and ionic liquid–polymer or ionic liquid–salt systems [21]. Additionally, ATPSs can be formed from proteins and polysaccharides [22,23,24,25], a category that has attracted increased attention due to discoveries that living cells naturally generate multiple membrane-less organelles (MLOs), also known as biomolecular condensates (BCs), through highly regulated biological liquid–liquid phase separation (LLPS) processes [26,27,28,29,30,31,32,33,34,35,36,37,38,39,40]. Importantly, cells represent highly sophisticated multi-phase systems, as they typically contain a very large number of different MLOs/BMCs [41,42,43].

## 3. Phase Diagrams as a Means to Describe ATPSs

**Figure 1:**
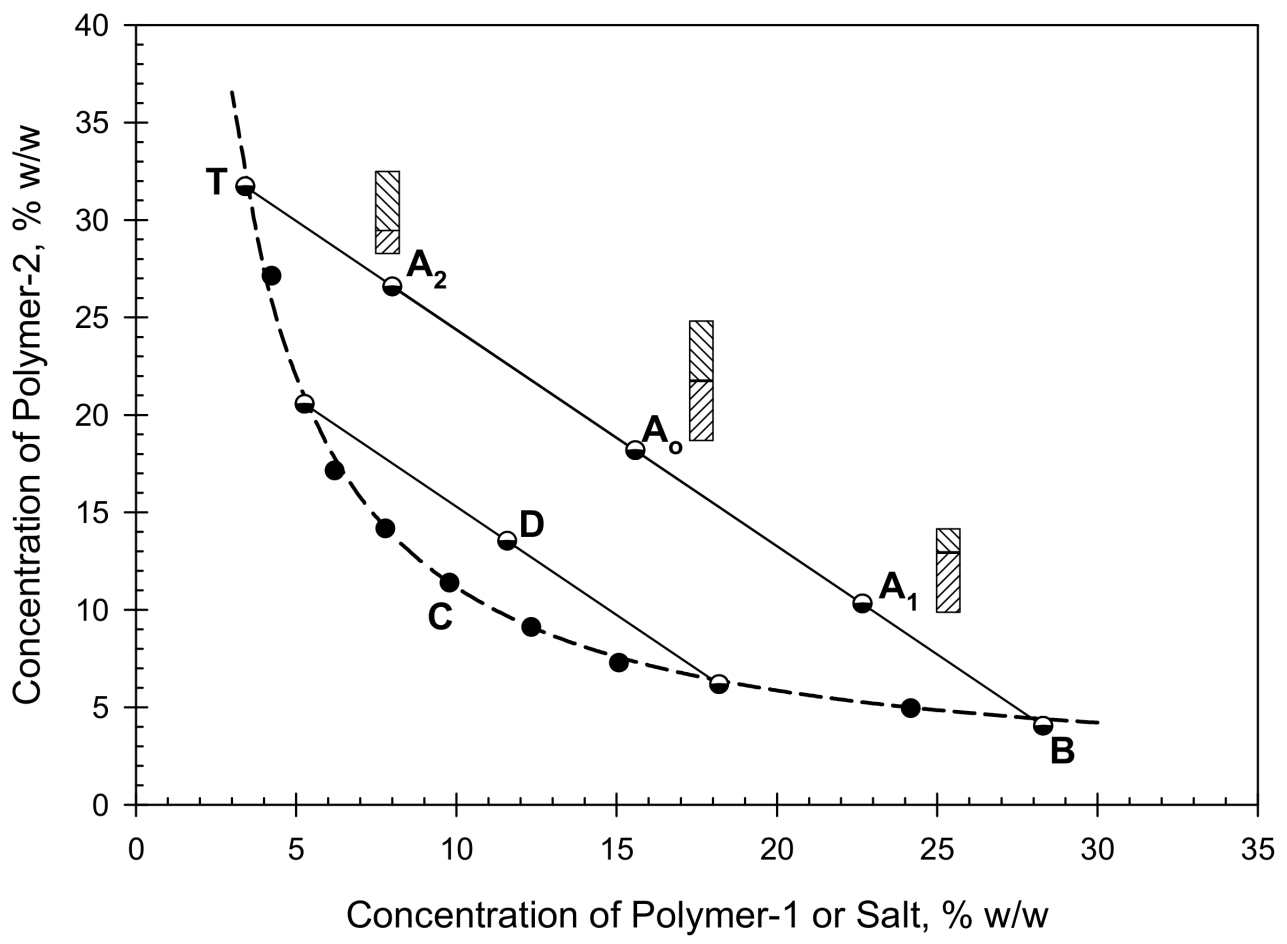
A typical aqueous two-phase system (ATPS) phase diagram illustrates the phase behavior of a water mixture containing polymer-1 (or a salt) and polymer-2. The system splits into a polymer-1 (or salt)-rich bottom phase and a polymer-2-rich top phase. The binodal line is a boundary curve that separates the two regions: the area under the binodal curve represents a single homogeneous liquid phase. The area above the binodal curve indicates the concentration range where the mixture splits into two distinct phases. The straight line connecting points T and B is a tie line. Every system prepared with a total composition along this line will separate into top and bottom phases with the exact same composition. Points A_o_, A_1_, and A_2_ represent different total initial compositions of the ATPS. Despite having different overall concentrations, they all fall on the same tie line and yield identical top and bottom phase compositions. Point T represents the specific chemical composition of the top phase. Point B represents the specific chemical composition of the bottom phase. Point C is the critical point, where the compositions and volumes of the two phases become identical.

The phase diagram in Figure 1 displays a curved boundary (binodal curve or coexistence curve) that demarcates two distinct regions and marks the thermodynamic limit of true miscibility, defining where a mixture separates into two distinct phases. Below this curve lies the single-phase region, where mixtures appear visually homogeneous. Above the binodal curve lies the two-phase region, where phase separation occurs. When a system’s composition falls within the two-phase region, it spontaneously separates into two coexisting phases, whose compositions are represented by the intersection points of the corresponding tie line with the binodal curve. The tie line connects the two phases’ compositions; indicates exactly what the concentration of the polymer, salt, and water will be in the top and bottom phases; and passes through the system’s overall composition point. As polymer (or polymer–salt) concentrations decrease, tie lines become progressively shorter, eventually converging at the critical point (labeled C in Figure 1), where the two phases become identical in composition [1,7,8,44,45].

A key feature of ATPSs is their compositional flexibility. Multiple systems can share identical phase compositions while differing in volume ratios [1,7,46]. For instance, points A_o_, A_1,_ and A_2_ in Figure 1 located on the tie line connecting two specific points on the binodal (saturation) curve represent different overall polymer compositions that all yield the same top phase (T) and bottom phase (B) compositions but with varying phase volume ratios. The length of the tie-line segments (i.e., T to A_o_ and A_o_ to B) can be used to determine the relative volume or mass ratio of the two resulting phases (lever rule). Conversely, compositions located off a given tie line (such as point D) will produce phases with entirely different polymer compositions. It is important to note that the tie-line length (TLL) dictates the behavior of ATPSs. This is because the shorter the tie-line length, the closer the system operates to the critical point on the binodal curve. As a result, in such systems, the solute partition coefficient is close to unity, since two phases are so similar compositionally (they have nearly identical compositions, densities, and physical properties) that solutes show no strong preference for either of them. Furthermore, near the critical point, the phase boundary (binodal curve) is highly sensitive to external variables. Small fluctuations in temperature can drastically shift the binodal curve, either merging the two phases into a single homogeneous phase or altering the phase volumes significantly. On the other hand, the longer the tie line, the further the system operates from the critical point. Here, the chemical and physical differences between the phases become highly pronounced, leading to a more extreme partition coefficient of a solute. Furthermore, being further away from the critical point, the two distinct phases are much more robust against minor temperature variations [1,7].

Note that phase separation in a single polymer solution with an invariant solvent composition can also be triggered by temperature. This temperature-induced phase separation (TIPS) occurs because of the temperature dependence of polymer solubility. It is characterized by the critical solution temperature, such as the upper critical solution temperature (UCST) in systems where cooling the solution decreases polymer solubility (i.e., the system mixes at high temperatures and separates upon cooling), or the lower critical solution temperature (LCST) observed in polymer–solvent systems, where heating the solution disrupts favorable polymer–solvent interactions (such as hydrogen-bonding) and drives demixing above a certain temperature [47]. Obviously, TIPS is characterized by a different phase diagram showing the temperature dependence of polymer solubility limits and defining when a homogeneous polymer solution splits into distinct polymer-rich and polymer-poor phases. Although TIPS represents a form of ATPS, consideration of such systems is outside the scope of this review.

ATPS preparation typically involves combining stock solutions of polymers and salts by mass. Since each component dilutes the others, careful calculation of stock solution concentrations is necessary to achieve the desired final composition. Stock solutions should be prepared gravimetrically. For hygroscopic polymers, like dextran and Ficoll, it is essential to prepare aqueous solutions first and then determine actual polymer content through freeze-drying or another suitable analytical method.

The binodal curve can be established through turbidimetric titration [48], while tie lines require analytical determination of polymer and/or salt concentrations in both phases. Unlike conventional organic solvent–water systems, a given polymer pair (or polymer–salt combination) can form numerous distinct two-phase systems with varying properties simply by adjusting the overall composition.

Polymer molecular weight significantly affects phase diagrams in both polymer–polymer and polymer–salt systems [7,49,50,51]. Generally, the binodal curve shifts downward as molecular weight increases, indicating that lower polymer concentrations suffice for phase separation at higher molecular weights. This trend supports the hypothesis that volume-exclusion effects drive phase separation [52].

On the other hand, the chemical composition of a polymer has been shown to be more critical than its size for phase separation. In identical buffer conditions (pH 7.4), dextran–PVP requires higher concentrations to phase separate than dextran–Ucon, despite PVP’s larger size (~12 kDa) compared to Ucon (~4 kDa). Similarly, in polymer–salt ATPSs [53,54,55], polypropylene glycol (PPG-725, 725 Da) forms two phases with sodium citrate at much lower concentrations than the larger PEG-4000 or Ucon-4000 (both ~4 kDa). Despite their equivalent molecular weights, the binodal curves of PEG-4000 and Ucon-4000 in sodium citrate mixtures occupy distinctly separate positions on the phase diagram. These observations demonstrate that chemical characteristics outweigh molecular size in determining phase separation behavior. These observations also hint at the important idea that phase separation might occur via the effect of solutes on the solvent properties of water (see below). The specific chemical and physicochemical characteristics that drive polymer phase separation in aqueous solutions are not yet completely understood.

Salt identity dramatically influences polymer–salt ATPS phase diagrams as well. For example, binodal curves for potassium versus sodium carbonate in PPG-400 solutions [55,56,57] differ markedly, as do those for sodium chloride versus sodium nitrate with PPG-425 [55,58].

Salt additives also affect phase diagrams in polymer–polymer systems, with effects studied more extensively in these systems (pp. 103–122, [7]) than in PEG–salt systems, although some examples exist [59,60,61]. The magnitude and direction of salt additive effects vary depending on the specific polymer pair, salt type, and concentration. The influence of salts on dextran–PVP and dextran–polyvinyl alcohol (PVA) phase diagrams is considerably more pronounced than that in dextran–PEG or dextran–Ficoll systems (pp. 103–116, [7]). Salt additions can alter phase volume ratios, signaling compositional changes even when binodal curves appear unchanged.

Interestingly, dextran and polyvinyl alcohol can form an ATPS in water even when completely miscible in the dry state (pp. 141–152, [7]). This observation validates the proposal [62] that aqueous polymer phase separation is driven by distinct impacts on water structuring, which leads to the formation of separate, immiscible hydrogen bond domains that retain their aqueous nature.

Phase diagram analysis serves two important purposes. First, it provides fundamental insights into phase separation mechanisms across different ATPS types. Second, it guides the rational optimization of conditions for protein and solute distribution. However, the accumulated phase diagram data has not yet provided sufficient mechanistic insight into phase separation processes.

Among the theoretical frameworks developed to explain phase separation in aqueous polymer mixtures, the binodal model introduced by Guan and colleagues [63,64] remains the most effective for accurately representing phase diagrams. This semi-empirical approach conceptualizes each point along the binodal curve as representing a saturated solution of one phase-forming component within a solution of the other component. While the original model relied on excluded volume principles, Ferreira et al. [65] proposed that the dissolution behavior of one compound in the solution of another may be governed by how that second compound alters water’s solvent characteristics.

Applying this modified binodal framework has yielded excellent results when describing phase diagrams for various polymer–polymer aqueous two-phase systems, including combinations of dextran, Ficoll, poly(ethylene glycol)-8000, and Ucon. The model has also successfully characterized a newly investigated system containing trimethylamine N-oxide (TMAO) and polypropylene glycol-400, as well as a previously documented TMAO and poly(ethylene glycol)-600 system. Additionally, it was demonstrated that this modified approach can be effectively applied to single polymer–salt and polymer–ionic liquid aqueous two-phase systems as well.

It should be stressed that, as discussed in detail by Zaslavsky in [7,66], the widely used Flory–Huggins approach and other theoretical approaches are not applicable to aqueous solutions. The key finding from our investigation is that phase separation in mixtures of various compounds (including polymers and salts) results from how these substances modify water’s solvent properties, thereby restructuring the water environment in their aqueous solutions. The manipulation of solute distribution is directly governed by the inherent properties of the coexisting phases. These traits must be evaluated for all ATPSs, regardless of whether complete phase diagram data exists. Ultimately, having a comprehensive phase diagram is not essential to understanding and controlling how a solute partitions.

## 4. Physicochemical Fundamentals of Aqueous Two-Phase Partitioning

Research has demonstrated that solute partitioning in ATPSs—ranging from small organic molecules to proteins and nucleic acids—generally occurs independently of direct interactions between solutes and phase-forming polymers, and that solute partitioning is primarily driven by changes in the solvent properties of the water in each phase. This conclusion has been proposed and subsequently validated through multiple studies [67,68,69,70,71,72,73]. However, in affinity partitioning or systems using charged polymers, this rule breaks down and specific polymer–solute interactions dictate partitioning. In fact, in affinity partitioning, ligands are specifically attached to one of the phase-forming polymers to selectively bind and extract a target solute) [74], whereas when polyelectrolytes are used in ATPSs, electrostatic interactions occur directly between the charged polymers and the solute [75].

The driving force behind unequal solute distribution in ATPSs is attributed to differential solute–solvent interactions between the coexisting phases. This phenomenon is primarily driven by dipole–dipole, dipole-induced dipole, hydrogen-bonding, and electrostatic interactions (including ion–ion, ion–dipole, and ion-induced dipole). By characterizing how these specific interactions affect a given solute within a particular ATPS, it is possible to predict its partition coefficient in a newly designed ATPS with known solvent properties at a 90% to 95% accuracy rate [67,68].

The strength and type of solute–solvent interactions depend heavily on solvent properties, although the exact impact varies based on the solute’s specific structure and chemistry. Polarity is the most common way to classify solvents, describing their ability to dissolve polar or ionic compounds. Currently, solvent polarity is understood to include all specific and non-specific interactions, such as electrostatic forces, dipole–dipole or dipole-induced dipole forces, hydrogen-bonding, and electron pair donor–acceptor interactions, excluding any interactions that chemically alter the solute.

Solvent polarity is a relative characteristic that defines how a solvent interacts with a solute, rather than a fixed, inherent property of the liquid itself. Because of this, there are multiple polarity scales derived from different analytical methods, including nuclear magnetic resonance (NMR), infrared (IR), and ultraviolet-visible absorption or emission spectroscopy. As highlighted by Ab Rani et al. [76], there is no single absolute metric for polarity. Every scale serves as an estimate, often producing varying values for the identical solvent. Ultimately, the value of an empirical polarity scale is not judged by whether it is “correct” but by how effectively it explains and predicts solvent-dependent chemical phenomena [76].

The solvent polarity of aqueous media in solutions of various ATPS-forming polymers (including dextran, Ficoll, and (PEG) of different molecular weights) was evaluated using a suite of water-soluble indicators. These included carboxylate-substituted anionic betaine (Reichardt’s) dye, thymol blue, sulfonephthalein derivatives, fluorescein, and eosin [77,78]. Research confirms that the solvent polarity of coexisting phases in dextran–Ficoll and dextran–PEG ATPSs exhibits distinct, measurable differences [67,68]. The differences in solvent polarity, hydrophobicity, and electrostatic characteristics between the phases [7] provide a foundation for comparing polymer–polymer ATPSs to organic-aqueous biphasic systems. Nevertheless, they differ critically in that both phases maintain an entirely water-based environment.

To better account for the diverse ways solutes and solvents interact, Kamlet and Taft introduced multi-parameter polarity scales through the Linear Solvation Energy Relationship (LSER), moving past the limitations of single-parameter scales. This approach incorporates three scales: (i) hydrogen-bond donor (HBD) acidity (α, which is a measure of a molecule’s ability to provide a proton (H^+^) to a hydrogen bond acceptor), (ii) hydrogen-bond acceptor (HBA) basicity (β, which refers to the thermodynamic tendency of a molecule or atom to act as a hydrogen bond acceptor), and (iii) dipolarity/polarizability (π*, which describe non-specific intermolecular interactions that define a solvent’s ability to stabilize a solute through electrostatic forces. Here, solvent dipolarity refers to the permanent dipole moment of solvent molecules that describes the solvent’s ability to orient its molecules around a solute to stabilize charge or dipoles through electrostatic (Coulombic) interactions. It is considered an anisotropic effect because it depends on the direction and orientation of the molecules. On the other hand, solvent polarizability measures how easily the electron cloud of a solvent molecule can be distorted by an external electric field (such as that from a solute). This distortion induces a temporary dipole, facilitating dispersion and induction forces. Being an isotropic effect, it acts uniformly in all directions regardless of molecular orientation [79,80,81]. The combination of these three scales provides a superior description of a solvent’s ability to participate in solute–solvent interactions compared to any single-parameter scale. They are used together because solute–solvent interactions are multi-dimensional and are influenced simultaneously by the medium’s charge stabilization, electron-donating ability, and electron-withdrawing ability. The LSER model [82] can be expressed by Equation (1):

XYZ = (XYZ)_0_ + sπ* + aα + bβ (1)

where XYZ is the solute property in a given solvent; (XYZ)_0_ is the same property in a reference state; s, a, and b are solute-dependent coefficients; and π*, α, and β are the respective solvent parameters.

The π* scale was generated by averaging values from seven solvatochromic dyes with strong, symmetric solvatochromic absorption spectra. To avoid dye-specific biases, multiple dyes were used to determine π*-values across more than 200 solvents, with 45 distinct dyes employed to date [83]. For aqueous media in polymer solutions and ATPS phases, the selection of water-soluble solvatochromic dyes is limited. For studying π*-values, 4-nitroanisole was selected [67] based on commercial availability, chemical stability, and water solubility.

As emphasized for ionic–liquid studies [76], the precise π*-value lacks fundamental physical meaning; rather, it serves as a guide to solvent effects on solute species sensitive to solvent dipole interactions. The magnitude of these effects must be evaluated within the appropriate physicochemical context. For example, while the π* difference between a 40% Ucon solution and a polymer-free control is 0.022, the corresponding variance between methanol and ethanol is 0.06; i.e., nearly three times greater. In contrast, for dextran-75 and Ficoll-70 ATPSs, the π* difference between coexisting phases is only 0.003. Despite this seemingly negligible difference, it still exerts a pronounced impact on the distribution of proteins and small solutes.

4-Nitrophenol is commonly used as the probe for β measurements, with β-values calculated from the wavelength of maximum absorbance and π*-value. It is important to note that, although in the context of the Kamlet–Taft solvent parameter model, the parameters β (hydrogen-bond acceptor basicity) and π* (dipolarity/polarizability) characterize fundamentally independent physicochemical properties, in practice, specific solvatochromic probes used to measure them are sensitive to both. For example, 4-nitrophenol interacts with the solvent via both general polarity (π*) and specific hydrogen bonding β). As a result, its absorption wavelength shifts due to a combination of both effects. Therefore, knowing the π*-value for a given solvent, one can mathematically subtract the solvent’s π* contribution to isolate the true β-value. The effects of the polymer on β are relatively small. The β-value difference between an aqueous 40% dextran-75 solution and a polymer-free medium is only 0.033. For context, this is much smaller than the 0.15 difference observed between methanol and ethanol. The β difference between the coexisting phases in a dextran-75–PEG-600 ATPS is even smaller, measuring just 0.005.

The water-soluble solvatochromic dye, sodium {2,6-diphenyl-4-[4-(4-carboxylato-phenyl)-2,6-diphenylpyridinium-1-yl]phenolate}, acts as a single probe for determining α-values, calculated from the maximum absorbance wavelength and π*-values. Data show that hydrogen-bond donating (HBD) acidity (α) decreases as polymer concentration rises. These polymer effects are striking: the α-value difference between polymer-free media and a 40% dextran-40 solution is 0.223, which is significantly larger than the 0.10 difference between methanol and ethanol. In ATPSs, α-value variations between coexisting phases range from 0.0 (for Ficoll-70 and PEG-6000) to 0.181 (for dextran-75 and Ucon), varying by polymer and salt composition. These results are consistent with previous findings regarding PEG’s impact on water’s HBD acidity.

Electrostatic interactions between a partitioned solute and the aqueous media in the phases heavily influence solute partitioning. Analyzing the partitioning behavior of a homologous series of dinitrophenyl (DNP) amino acid sodium salts with growing aliphatic alkyl side chains (glycine, alanine, norvaline, norleucine, and α-amino-n-octanoic acid) helps characterize the electrostatic differences between phases. Equation (2) describes the partition coefficient logarithms for these compounds:

log K^DNP-AA^_i_ = C^i^ + E^i^ N_c_ (2)

where K^DNP-AA^_i_ represents the partition coefficient of the DNP–amino acid sodium salt within the *i*-th ATPS. The side chain’s overall hydrophobicity and length are quantified by N_c_, which matches the equivalent number of straight-chain CH_2_ groups. The characteristics of the *i-*th ATPS are defined by the constants *E* and *C*. Here, the parameter *E* represents the average logK per CH_2_ group, which directly converts to the free energy of transfer between phases. This value highlights the difference in phase hydrophobicity. For context, E ≈ 0.50 in octanol–water systems but ranges from roughly 0.005 to 0.12 in polymer–polymer systems. The parameter *C* reflects the specific contribution of the polar component, which is the DNP-NH-CH-COONa moiety. While this moiety serves as a probe for electrostatic properties, its specific advantages and limitations are well-documented. Ultimately, adding different additives alters the solvent properties of standard polymer–polymer and polymer–salt systems, which directly influences how proteins partition.

Solvent dipolarity/polarizability (Δπ*) differences vary within similar ranges in both dextran–PEG and PEG–Na_2_SO_4_ ATPSs, as do electrostatic property differences (C). Hydrogen-bond basicity (Δβ) differences are typically larger in PEG–Na_2_SO_4_ ATPSs compared to dextran–PEG ATPSs, a pattern also observed for hydrogen-bond acidity (Δα). The only universal rule applicable to proteins and other soluble compounds is that for K > 1, the partition coefficient increases with increasing phase polymer (or polymer and salt) concentrations, whereas for K < 1, the partition coefficient decreases under similar conditions. In an ATPS, solute partitioning, including that of proteins, is independent of molecular size and weight. Rather, it is governed exclusively by the chemical nature and spatial organization of the functional groups exposed to the surrounding solvent.

## 5. Characterization of Individual Proteins with Solvent Interaction Analysis

Experimental evidence [84] shows that two peptides sharing identical amino acid compositions but differing in sequence can exhibit partition coefficients that vary more than threefold. The magnitude of these differences, as well as the actual partition coefficient values for small organic molecules, can fluctuate significantly depending on the specific polymer and ionic composition of the employed ATPS.

As mentioned earlier, the partition coefficient characterizes a solute’s partition behavior and is calculated as the solute concentration ratio between the top and bottom phases. It has been demonstrated that a compound’s partition behavior in an ATPS is extremely responsive to structural modifications of a solute [7,51,67,69,70,85,86,87,88,89]. Table 1 further demonstrates this by showing the partition coefficients across various mutants of bacteriophage T4 lysozyme and staphylococcal nuclease A.

Table 1 also contains information about the structural signatures (or SIA signatures) of the analyzed proteins. The SIA signature is a precise numerical fingerprint created by combining the partition coefficients (K-values) from a single protein analyzed across various ATPSs. This approach organizes the resulting partition coefficients into a single vector. The vector functions as a highly sensitive signature capable of detecting structural anomalies and molecular shifts, such as single-point mutations, misfolding, aggregation, and interactions with binding partners [89,93,94]. Mathematically, the SIA signature quantifies structural changes by calculating the normalized Euclidean distance between a query form of a protein and a reference (here, the wild type of the same protein under evaluation). These distances are derived from the logarithms of the partition coefficients using Equation (3):

(3) $$d_{i,0}=\;\sqrt{\sum_{j=1}^{n}{\left(\frac{logK_{i,j}-logK_{0,j}}{logK_{0,j}}\right)}^{2}\;\frac{s_{j}}{s_{max}}}$$

where the distance d_i,0_ is determined between the *i*-th mutant signature and the reference signature across *n* aqueous systems (which is three in this study). The subscript *j* refers to the *j*-th ATPS, where S_j_ is the normalized standard deviation in system *j* and Smax is the maximum standard deviation observed across all utilized systems. Research indicates that SIA signatures derived from protein partitioning in ATPSs serve as straightforward numerical descriptors that capture the structural and dynamic properties of query proteins. These parameters reflect the interactions between a solute’s surface and the aqueous solvent, making them highly sensitive to protein mutations, post-translational modifications, structural dynamics, and overall conformational stability [89,93,94,95]. It is important to note that although the SIA signature relies on the thermodynamic, hydrogen-bonding, and electrostatic interactions with the hydration shell, it does not represent an absolute property, as it detects changes in the protein structure that occurred relative to that in the reference sample.

Table 1 presents an example of the impressive sensitivity of this approach. Protein partitioning has additionally been demonstrated to vary based on the nature and spatial organization of chemical groups accessible to the solvent [96,97].

To determine an individual protein’s partition coefficient, the recommended procedure [98] involves preparing multiple identical polymer and salt additive mixtures at concentrations above those desired in the selected ATPS. A “free volume” of water or sample solution must then be incorporated to reach the target polymer and salt composition. This free volume typically ranges from 50–250 μL for total ATPS volumes of approximately 400–1500 μL, depending on the specific ATPS composition. To achieve varied solute concentrations within the ATPS, different volumes of solute solution combined with corresponding water volumes (totaling the free volume) can be added.

**Figure 2:**
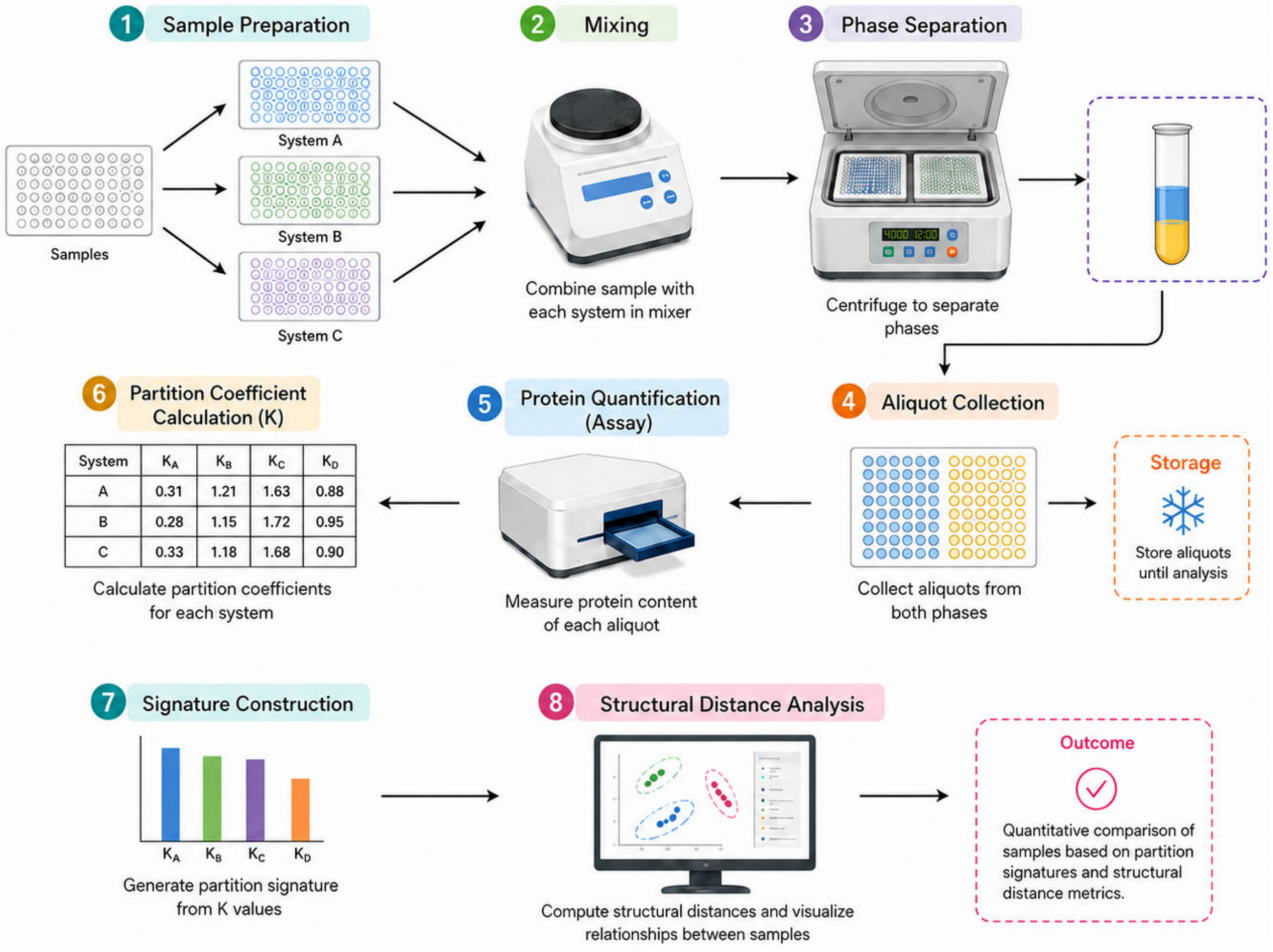
Schematic representation of the solvent interaction analysis (SIA) process. The SIA process consists of two primary operational stages: the SIA assay and the analytic assay. The initial stage focuses on partition sampling and preparation through the following steps: (1) Sample preparation: Aliquots of the target sample are introduced into distinct ATPSs, labeled Systems A, B, and C, each featuring a unique ionic composition. (2) Mixing: The mixtures undergo thorough mechanical agitation. (3) Phase separation: Centrifugation is used to accelerate the separation of the phases. (4) Aliquot retrieval and dilution: Samples are drawn from both the top and bottom phases and diluted appropriately. This step can be executed manually or automated via a liquid-handling workstation; the collected aliquots are either transferred immediately to the next stage or stored long-term in a refrigerator or low-temperature freezer. The second stage translates the physical samples into analytical structural data using the following steps: (5) Analyte quantification: The precise concentration of the analyte within each aliquot is determined using a compatible bioanalytical method. Suitable approaches include high-performance liquid chromatography (HPLC), liquid chromatography–mass spectrometry (LC-MS), various mass spectrometry techniques, single or multiplexed immunoassays, or protein quantification assays like Bradford or o-phthalaldehyde (OPA). (6) Partitioning analysis: The partition coefficient (K) is computed for each distinct ATPS composition. (7) Signature generation: A definitive structural signature for the analyte is established by compiling and plotting its K-values across the different systems. (8) Comparative evaluation: The structural distance is calculated between the analyte’s generated signature and the signature of a designated reference standard.

Following solute addition, the systems undergo mixing and centrifugation to accelerate phase separation. Aliquots are then collected from the separated phases, diluted as necessary, and analyzed using appropriate concentration assays. This workflow can be performed manually in 2 mL vials (see the video demonstration at www.youtube.com/watch?v=0h9bTSO7Zv4; accesses on 24 July 2026), using microtubes or deep-well plates with liquid-handling workstations [99,100,101,102], or in microchip formats [103,104,105,106,107,108,109,110,111,112,113,114]. Figure 2 provides a graphical representation of the complete process.

Various analytical techniques can measure protein concentrations in the diluted phase aliquots [100]. Available methods include HPLC (sensitive yet relatively time-intensive); mass spectrometry/MS (rapid and sensitive, although requiring internal standards for concentration determination); o-phthaldialdehyde/OPA assay (fast, dependable, and highly sensitive for proteins containing adequate lysine residues); Bradford assay (straightforward and quick, albeit less sensitive than OPA); UV absorbance measurements (generally requiring larger protein quantities due to limited sensitivity); and ELISA or similar affinity-based assays (highly sensitive and suitable for analyzing specific proteins in biological fluids including serum, plasma, urine, cerebrospinal fluid/CSF, tissues, or cell extracts). Calibrating the analytical method in diluted phases from both top and bottom is advisable, although not invariably essential.

**Figure 3:**
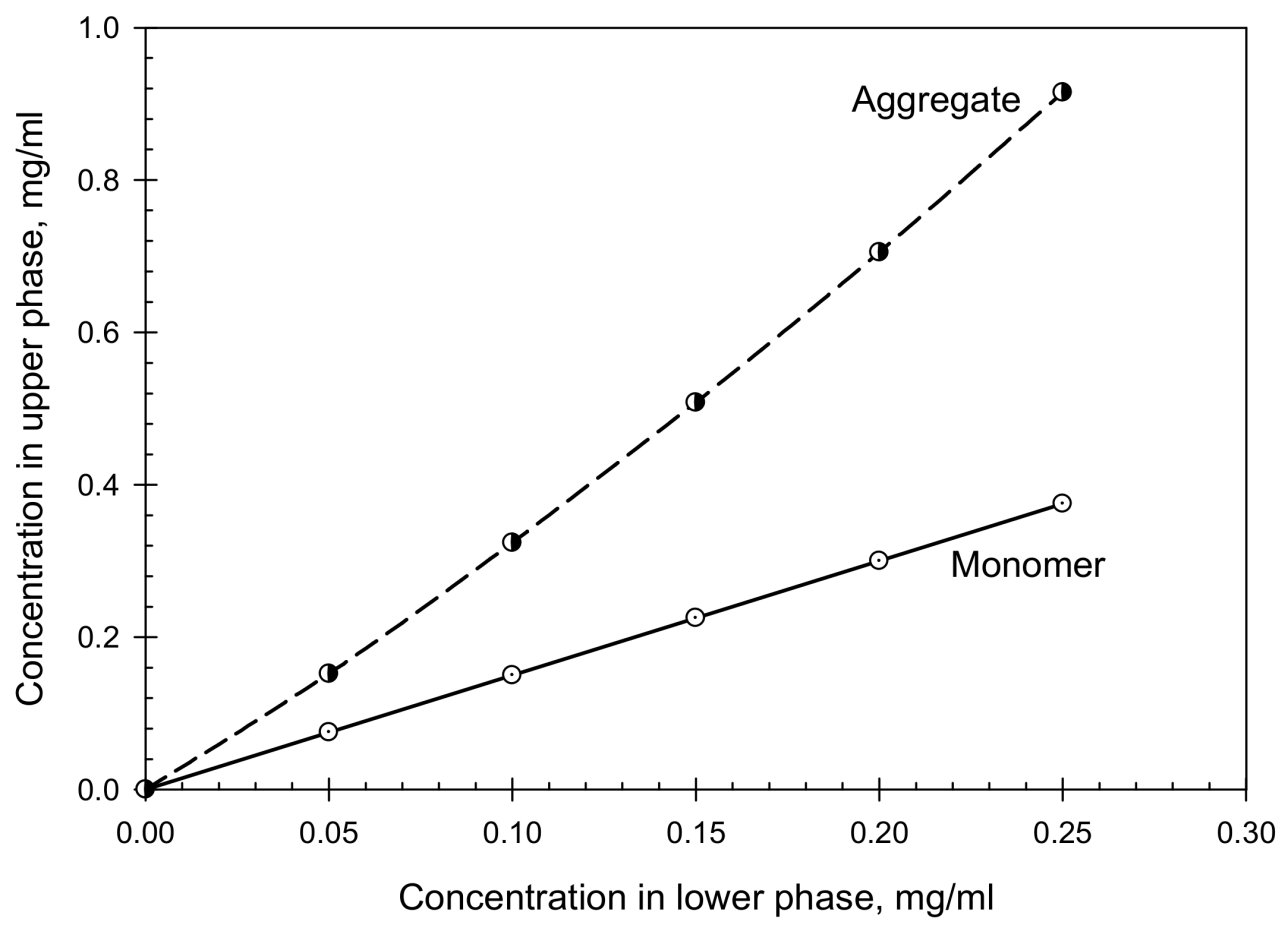
Protein concentrations determined in the upper phase plotted against protein concentrations determined in the lower phase. For monomeric proteins, the distribution between the phases is uniform and proportional. This results in a straight line in the plot, the slope of which directly represents the partition coefficient. For aggregating proteins, as the total concentration of the protein increases, individual monomers begin to self-associate and form dimers, trimers, or larger oligomeric aggregates, which possess different surface properties, sizes, and hydrophobicities compared to single monomers and, therefore, partition drastically differently from the individual units. Because the ratio of aggregate to monomer changes as more protein is added, the overall partition ratio shifts. This causes the plot to bend into a nonlinear curve. Therefore, deviation of the plotted curve from linearity indicates protein aggregation.

Upon withdrawal and dilution, phase aliquots may be refrigerated or frozen before measuring protein concentration. The protein partition coefficient is computed based on the protein concentrations or their proportional analytical signals. During this determination, the analytical values recorded for the top phases are plotted against the corresponding bottom-phase values (see Figure 3 for a representative plot). The slope of the linear plot defines the protein partition coefficient, while the y-intercept reflects background interference from phase components on the analytical signal, which frequently approaches zero. The plot’s linearity indicates that the protein partition coefficient K remains independent of protein concentration, implying identical species distribute between the coexisting phases across the experimental protein concentration range. Deviation from linearity between upper and lower phase solute concentrations suggests that elevated protein concentrations can generate species exhibiting partition behavior distinct from that of species present at lower concentrations, typically indicating protein aggregate formation. The nonlinear curve in Figure 3 illustrates typical protein aggregation behavior. Measuring K-value partition coefficients typically yields an accuracy better than 3%. However, this precision varies by the chosen analytical technique, with immunoassays often showing significantly higher margins of error. The optimal target protein concentration depends on the assay’s detection limits and the specific testing goals. For diagnostic applications, economic and throughput considerations typically necessitate using single sample volumes.

The complete protein partition analysis in an ATPS involves two main steps: partitioning the protein within the system and measuring its concentration in each separated phase. After phase separation and dilution, samples can be stored at low temperatures before final analytical assay preparation.

For proteins, the partition coefficient depends on the polymer composition of the ATPS. While extreme protein distribution is ideal for protein isolation, analysis and characterization require a partition coefficient (K) within an analytically reliable range of 0.1 to 10. More extreme distributions are difficult to measure accurately because the protein concentrations between the two phases differ by more than an order of magnitude. Generally, ATPSs composed of dextran-70 (70 kDa) or dextran-40 (40 kDa) combined with Ficoll-70 (70 kDa), or Ficoll-70 combined with PEG (600 to 8000 Da), are well-suited for protein analysis, although PEG–salt systems can also be used.

Once an ATPS with a polymer composition that yields protein partition coefficients within the target range (0.1 < K < 10) is identified, the system must be fine-tuned to meet specific analytical objectives. This objective could involve detecting protein sequence alterations (like mutations or splicing), post-translational modifications (such as glycosylation or phosphorylation), conformational shifts, partner binding, or chemical modifications (like oxidation or deamidation). In these cases, the goal is to optimize conditions so that the differences in K-values between the intact and modified proteins are large enough to ensure analytical reliability. The most effective way to achieve this is by manipulating the ionic composition of the ATPS.

Table 2 presents examples of different K-values for human serum albumin when exposed to various concentrations of different drugs [115].

It is worth noting that the data from [115] shown in Table 2 were generated with the objective of exploring conformational changes in the protein induced by the binding of drugs.

The data listed in Table 2 show that the ratio of the partition coefficient of albumin at various drug concentrations on a logarithmic scale (lnK_ij_) to the partition coefficient of HSA in the absence of the drug (lnK_0_) may be described by Equation (4):

Ln(K_ij_/K_o_) = A + B exp(C[drug]_j_), (4)

where A, B, and C are constants. These constants reflect the drug’s effect on the conformational change of HSA.

The clearance times for the drugs investigated in [115] are known [116], and the analysis showed that there is a linear relationship that can be described by Equation (5):

exp(-CT) = −0.42 _± 0.06_ + 0.20 _± 0.03_1/C + 0.33 _± 0.04_exp(-B) (5)

N = 10; r^2^ = 0.920; SD = 0.097; F = 40

where CT is the drug clearance time.

These results showed that HSA’s partitioning behavior in the ATPS is influenced by its interactions with various drugs in a manner that depends on drug concentration. While all examined drugs increased the HSA partition coefficient, each affected protein partitioned differently, with the concentration-dependent relationship for each drug following an exponential pattern. These findings suggest that all tested drugs alter HSA conformation. The relationship between the exponential equation parameters that describe HSA partitioning in the presence of a specific drug and that drug’s clearance rate from the human body implies that drug-induced conformational changes in HSA, which are reflected in how the HSA–drug complex partitions in ATPSs, are connected to the drug’s blood clearance efficiency. These findings also demonstrate the practical value of using protein partitioning in ATPSs as a useful analytical tool for diverse applications.

Next, we explain the rationale behind the name solvent interaction analysis (SIA). Research by Madeira et al. [67,68,69,86,88,117], Ferreira et al. [71,118,119], and da Silva et al. [72,120] confirms that Equation (4), when expanded to include an electrostatic interaction parameter, successfully models the partition coefficients of proteins and small organic compounds. This holds true for ATPSs composed of different polymer pairs with identical ionic compositions, as well as polymer–salt ATPSs with a fixed ionic composition. Crucially, these studies established that the contributions of specific solute–solvent interactions (such as dipole–dipole, electrostatic, and hydrogen-bonding) depend entirely on the solute itself. They remain completely unaffected by the type, molecular weight, or concentration of the phase-forming polymers or any nonionic additives that alter phase solvent properties [67,68,69,71,72,86,88,117,118,119,120]. These findings confirm earlier suggestions [7] that solutes interact exclusively with the water in the two phases rather than directly with the phase-forming polymers. Because the properties of this aqueous environment can be altered using additives like osmolytes [71,72,118,119,120], the distribution of proteins between the phases also shifts. Consequently, by measuring how a protein partitions across the two phases, we can evaluate the differences in its solvent interactions, a method known as solvent interaction analysis (SIA).

It has been thought that the partition of proteins in ATPSs is related to the protein molecular weight [52]. Ferreira et al. [121], however, demonstrated that trimethylamine N-oxide (TMAO), a nonionic additive, can be used to control how solutes distribute in aqueous two-phase systems. The researchers investigated the partitioning behavior of various proteins and small organic molecules in two different aqueous two-phase systems: one containing dextran-75, Polyethylene glycol (PEG-600), 0.15 M sodium sulfate, and 0.01 M sodium/potassium phosphate buffer at pH 7.4, with TMAO concentrations ranging from 0 to 1.95 M; and another consisting of Ficoll-70, Polyethylene glycol (PEG-8000), and 0.01 M sodium/potassium phosphate buffer at pH 7.4, with TMAO concentrations ranging from 0 to 1.5 M. The study revealed that protein partitioning responds to increasing TMAO concentrations in a protein-dependent manner. To characterize how the solvent properties of water differ between phases—including hydrophobicity, electrostatic characteristics, solvent dipolarity/polarizability, hydrogen-bond donor acidity, and hydrogen-bond acceptor basicity—the researchers employed two approaches: examining the partitioning patterns of homologous sodium salts of dinitrophenylated amino acids containing aliphatic alkyl side chains and applying the solvatochromic comparison technique. The findings indicated that TMAO influences solute partitioning by modifying the solvent characteristics of each phase, supporting the hypothesis that the distribution of proteins and small organic molecules is determined by solute–water interactions within the coexisting phases.

**Figure 4:**
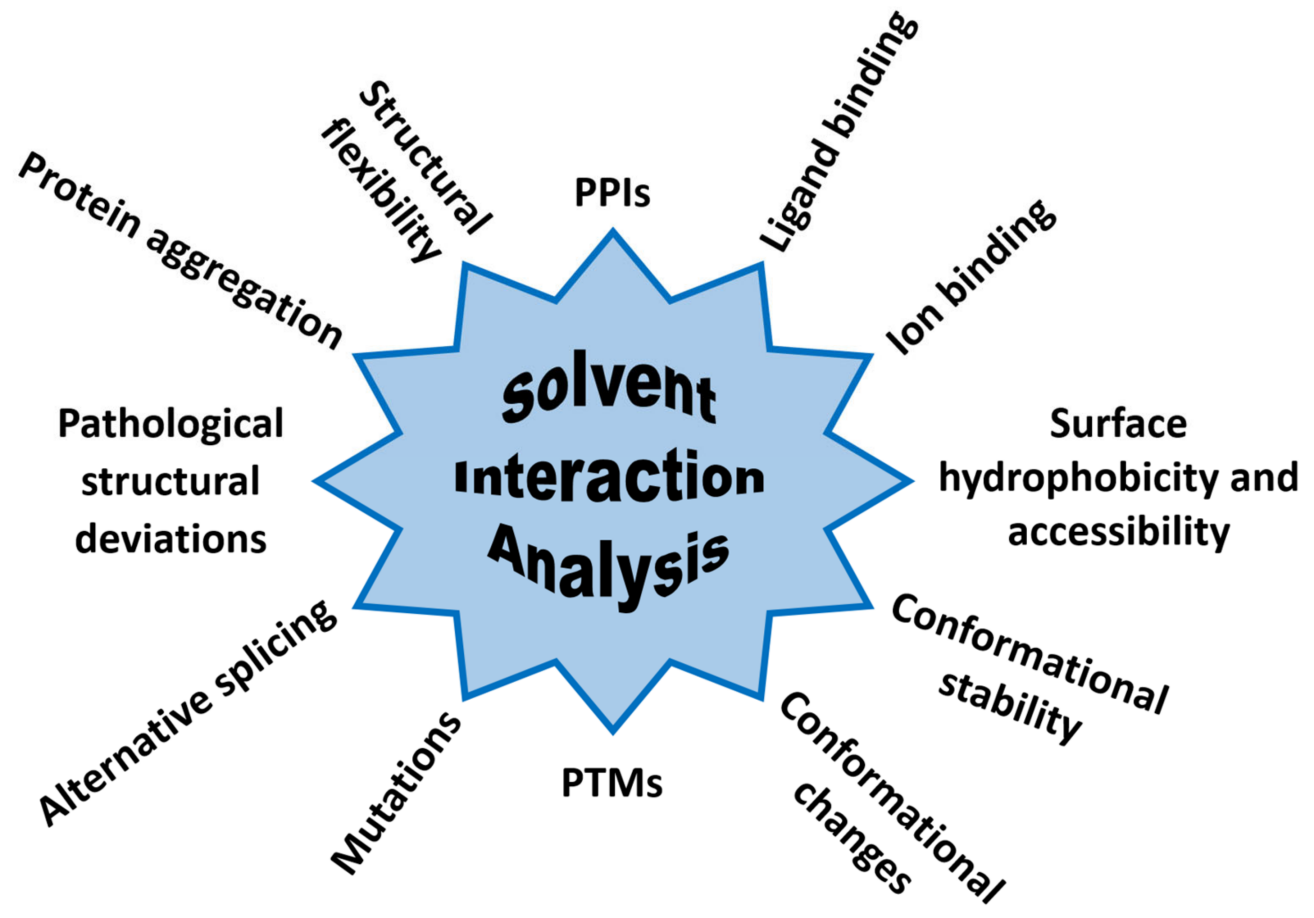
Key protein characteristics that ATPS-based SIA can distinguish and analyze.

Figure 4 summarizes some of the major advantages of the application of ATPS-based SIA to proteins and shows that this technique represents a unique tool for characterizing various features ranging from 3D conformation to structural dynamics, interactions, and post-translational modifications. By measuring the peculiarities of the partition of proteins between two immiscible aqueous phases, it also represents a powerful label-free analytical platform to separate, identify, and characterize the diverse molecular forms (proteoforms) generated by genetic variations or modifications (like phosphorylation or glycosylation), which are notoriously difficult to separate using conventional methods.

## 6. Structure-Based Proteomic Approaches for Biomarker Discovery

Proteomics, the large-scale study of protein structures and functions, is much like space exploration: both fields expand our understanding of their respective universes while driving the creation of innovative technologies. In practical terms, proteomic research fuels medicine by discovering new biomarkers and drug targets. However, a significant gap exists between biomarker discovery and clinical application. While researchers identify numerous potential biomarkers [122,123,124,125,126,127,128,129,130], only a small fraction is validated for laboratory use [131,132,133,134,135,136,137,138,139,140,141,142]. In fact, fewer than 1.5 protein biomarker tests were approved annually for clinical practice over a 16-year period [143]. Broadly defined, a biomarker is any measurable bodily substance, structure, or process that predicts or influences health outcomes and disease progression [144]. Specifically, a protein biomarker is a protein that appears, alters, or is excreted by the body as a direct biological response to a disease.

Different types of natural compounds may be considered potential disease biomarkers. Proteins offer distinct advantages as potential disease biomarkers due to their vast diversity, dynamic turnover, and presence in secreted biological fluids. When accounting for post-translational modifications, the sheer number of unique proteoforms exceeds the total count of genes, metabolites, and mRNA transcripts by more than an order of magnitude. This immense variety significantly improves the likelihood of identifying specific markers or multi-marker panels for distinct disease states [134]. Because the primary objective of biomarker discovery is typically to create a blood-based test, blood serves as the most logical source material. Human plasma, in particular, is considered the most comprehensive representation of the human proteome because it contains components from all body tissues reflecting both normal and diseased processes. This completeness, combined with the ease of collecting plasma and the extensive medical laboratory infrastructure already equipped to analyze it, ensures that plasma will remain the primary diagnostic material for the foreseeable future.

Proteins within biological fluids alter their properties during disease states. Current techniques enable the measurement of three key changes: concentration levels, structural variations (e.g., proteoform ratios, truncations, mutations, and post-translational modifications), and functional dynamics (e.g., protein interactions with other proteins, nucleic acids, or small molecules) [145].

The search for protein biomarkers relies heavily on measuring their concentrations in biofluids like serum, plasma, urine, or cerebrospinal fluid (CSF). This established strategy represents a long-established trend that persists for two primary reasons. First, human pathologies and physiological changes are fundamentally reflected by fluctuations in or the presence of specific proteins in these fluids [146,147]. Second, affinity-based measurement technologies are highly accessible and continuously evolving [148,149,150,151,152,153,154,155,156,157,158]. Consequently, modern clinical chemistry laboratories still depend almost entirely on these affinity-based assays to quantify biomarkers in patient samples.

Although measuring total biomarker concentration is a historically established diagnostic approach, it may not be the optimal strategy [146]. This method often groups distinct proteoforms under a single denominator [159,160,161]. As Bults et al. noted [159], measuring a “protein” is only meaningful if the specific method and the exact molecular property it detects are clearly defined. To address this issue, mass spectrometric immunoassay (MSIA) can be used to quantify different plasma protein proteoforms [162,163,164,165,166,167,168,169,170,171,172,173,174,175,176]. However, inter-individual variability in plasma and serum protein levels presents an even greater diagnostic challenge. For instance, Liu et al. systematically studied this variability by analyzing 342 proteins across 232 longitudinal plasma samples collected from healthy monozygotic and dizygotic twins over 2–7 years [177]. Using SWATH-MS, a high-throughput targeted mass spectrometry method [178,179,180], they discovered substantial baseline variation between individuals in 174 of the examined proteins [177]. This high variability was especially prominent in low-abundance proteins, which are precisely the molecules most frequently pursued as promising disease biomarkers [177].

Protein expression levels in blood are highly dynamic and can change significantly in response to normal physiological conditions, making them unreliable as diagnostic markers when considered alone. Vernardis et al. [181] showed that short-term caloric restriction and refeeding altered more than 10% of abundant plasma proteins, including proteins previously proposed as disease biomarkers. These changes reflected normal metabolic adaptations rather than pathology. Similarly, sleep deprivation caused substantial alterations in hundreds of plasma proteins, affecting pathways related to inflammation, coagulation, oxidative stress, and immune function [182,183]. Exercise training also changed the expression of 453 plasma proteins in healthy individuals, with most showing increased levels [184]. In addition, lifestyle factors such as smoking, alcohol consumption, and physical activity were associated with changes in dozens of circulating proteins [185]. These findings demonstrate that protein abundance is influenced by numerous physiological and environmental factors, reducing the specificity of individual protein levels for disease diagnosis.

Clinical chemistry traditionally relies on a single, population-wide threshold to detect disease based on whether a biomarker concentration falls outside a standard reference range. However, analysis of longitudinal changes in cancer antigen 125 (CA-125) levels in ovarian cancer patients suggests that this population-wide approach is suboptimal [186], largely due to high inter-individual variability [187]. While CA-125 is clinically used to monitor treatment efficacy and detect recurrence, it is not recommended for general screening because it misses approximately 50% of early-stage ovarian cancers. Conversely, Drescher et al. demonstrated that CA-125 can effectively screen for ovarian cancer when evaluated longitudinally [186]. By conducting annual serial measurements, each patient establishes an individual baseline. Collectively, these findings indicate that static plasma protein concentration alone may not be the most effective diagnostic metric [186].

Structural variation is a vital aspect of protein biomarkers, as proteins typically exist in plasma as families of proteoforms. Ratios of these variant forms have long been used in diagnostics. For instance, serum transferrin presents as multiple proteoforms varying in sialic acid content. Sustained heavy alcohol use can elevate the fraction of transferrin with reduced sialic acid by up to 15-fold; this specific variant is known as carbohydrate-deficient transferrin (CDT) [188]. Blood serum ratios of CDT to total transferrin serve as standard clinical biomarkers for diagnosing congenital disorders of glycosylation (CDG) and chronic alcohol misuse [189]. Furthermore, comparing serum levels of glycated albumin to glycated hemoglobin (HbA1c) provides strong insights into insulin function [190]. In type 1 diabetes, this ratio acts as a more accurate measure of glycemic variability than relying solely on HbA1c [191]. The idea that relying on protein isoform ratios rather than absolute concentrations offers highly reliable diagnostic biomarkers is further supported by the observation that the ratio of platelet amyloid precursor protein isoforms serves as a promising indicator for presymptomatic Alzheimer’s disease [192]. Furthermore, Creutzfeldt–Jakob disease is distinguished from other dementias using the ratio of phosphorylated to total tau proteins in cerebrospinal fluid [193].

Mass spectrometry allows researchers to measure different proteoforms within biological fluids [162,163,164,165,166,167,168,169,170,171,172,173,174,175,176]. To understand a disease, scientists must identify which specific proteoform traits or features drive the condition. Protein function depends heavily on structure, which post-translational modifications (PTMs) can alter within milliseconds in response to cellular stimuli [194]. Glycosylation is the most stable PTM, affecting over half of all human proteins. Mass spectrometry of these protein-bound carbohydrates [195,196,197,198,199,200,201] has linked specific glycosylation patterns to various diseases [201,202,203,204,205]. For instance, a glycomic analysis of serum from 52 women with low malignant potential tumors, 147 with epithelial ovarian cancer, and 100 healthy controls revealed distinct, diagnostically promising variations in glycan composition [206].

Research highlights distinct changes in protein glycosylation profiles for several conditions. In ovarian cancer, studies report altered glycosylation profiles (relative to those of healthy women) for CA-125 [207], haptoglobin [208,209], α1-acidglycoprotein and α1-antichymotrypsin [208], clusterin, complement factor H, and hemopexin [210], leucine-rich alpha-2-glycoprotein [211], and other glycoproteins [212,213]. Similarly, significant plasma glycosylation differences distinguish various breast cancer types from controls [214,215,216,217,218,219,220,221]. Furthermore, patients with pancreatic cancer show significant shifts in the glycosylation of proteins, such as α1-acid glycoprotein, α1-antitrypsin, fetuin, haptoglobin, transferrin [222,223], ceruloplasmin [224], thrombospondin-1 [225], alpha-2-macroglobulin [226], and certain other proteins [227,228,229,230] compared to individuals with chronic pancreatitis or healthy controls. Glycosylation variations occur in colorectal [231,232,233,234], gastric [235], liver [236,237,238,239,240], lung [241,242,243], brain [214], and prostate cancers [244,245,246,247], as well as melanoma [248], schizophrenia [249], and other diseases. While integrating comprehensive glycomic analysis into clinical practice remains difficult, utilizing the SIA method offers a simpler alternative by tracking changes in glycoprotein partition coefficients to reflect these altered glycosylation patterns.

Therefore, SIA represents a powerful, structure-focused approach to clinical diagnostics that shifts the focus from simple bulk protein abundance to the precise structural and functional states of protein biomarkers. By directly probing how proteins interact with their liquid environment, SIA provides critical, real-time insights into disease pathology that traditional concentration-based assays miss. The section below illustrates the power of this technology in clinical settings.

## 7. IsoPSA as an Illustration of the Power of SIA

Prostate-specific antigen (PSA)-based screening has substantially improved the early detection of prostate cancer (PCa); however, it is limited by poor specificity for clinically significant disease, particularly within the 4–10 ng/mL “gray zone,” where elevated PSA levels are frequently attributable to benign prostatic conditions rather than malignancy [250,251,252,253,254,255]. Consequently, contemporary clinical guidelines recommend a shared decision-making approach to PSA screening in average-risk men aged 55–69 years, emphasizing the need to balance the potential reduction in prostate cancer-specific mortality against the risks of overdiagnosis and overtreatment associated with indiscriminate biopsy and treatment [255,256,257]. Collectively, these limitations highlight a critical unmet need for a blood-based biomarker capable of more accurately identifying patients harboring actionable, high-grade prostate cancer.

**Figure 5:**
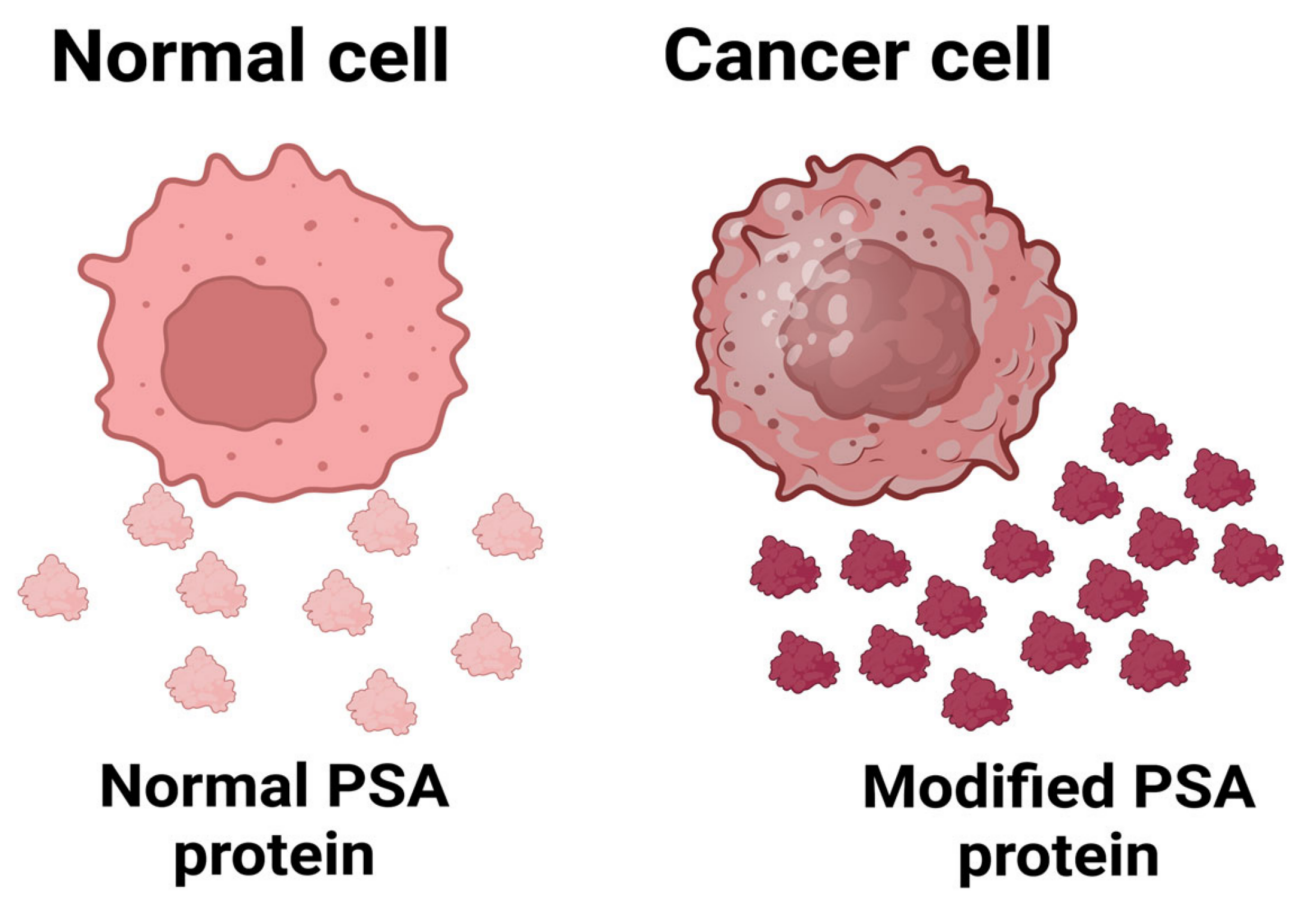
Illustration highlighting the quantitative and qualitative differences in PSA protein secreted by healthy versus malignant cells. Aberrant PSA is directly linked to the development of several biological networks in cancer, most notably androgen receptor (AR) signaling, the kallikrein (KLK) proteolytic cascade, and angiogenesis and growth factor pathways. PSA dysregulation occurs not through direct mutations but as a downstream effect of genetic and epigenetic alterations in these pathways [263,264,265,266,267].

At the molecular level, malignant transformation of prostate epithelium is associated with alterations in PSA isoforms, including post-translational changes in glycosylation patterns, protein conformation, and intermolecular interactions, relative to PSA derived from benign tissue [244,246,247,258,259] (see Figure 5). The IsoPSA assay, which instead of measuring the total amount of PSA in the blood analyzes the structural changes (isoforms) of the protein, is designed to detect these structural differences using an aqueous two-phase partitioning system, generating a ratiometric index that reflects the composite physicochemical properties of circulating PSA [95]. This index has been shown to discriminate high-grade prostate cancer (HG-PCa; Gleason score ≥ 7/Grade Group ≥ 2) from low-grade disease (LG-PCa) and benign histopathology on biopsy, using the proprietary IsoClear Platform (Cleveland Diagnostics, Inc.; Cleveland, Ohio, USA) [260,261,262]. The clinical value of IsoPSA in informing biopsy decision-making is grounded in the well-established limitations of total PSA, particularly within the gray zone, where confounding factors such as benign prostatic hyperplasia, inflammation, and pharmacologic influences can significantly impair specificity [255,256,257].

These factors limit the ability of total PSA to reliably distinguish HG-PCa from LG-PCa or non-malignant conditions. In parallel, multiparametric Magnetic Resonance Imaging (mpMRI), while an important adjunctive tool, demonstrates reduced diagnostic certainty in patients with PI-RADS 1–3 findings, where false-negative, indeterminate, and false-positive results remain clinically relevant limitations [268].

IsoPSA addresses these diagnostic challenges through a distinct and mechanism-based approach. Rather than quantifying total PSA concentration or targeting predefined molecular isoforms, the assay evaluates cancer-associated structural alterations in PSA in an agnostic manner. Malignant prostate cells produce PSA with altered glycosylation profiles, conformational states, and protein-binding characteristics, which collectively influence its partitioning behavior and are captured by the IsoPSA index [95,244,246,247,258,259].

In a prospective, multicenter clinical validation study of men aged ≥ 50 years with total PSA ≥ 4 ng/mL undergoing diagnostic biopsy, IsoPSA achieved an area under the receiver operating characteristic curve (AUC) of approximately 0.78 for the detection of high-grade prostate cancer [260]. At prespecified IsoPSA index cutoffs, the assay maintained high sensitivity for HG-PCa while enabling an estimated reduction of up to about 45% in unnecessary biopsies among men at low risk, reflecting favorable tradeoffs between sensitivity, specificity, and the proportion of avoided biopsies.

Longitudinal data further supports the prognostic relevance of baseline IsoPSA values. In a cohort of men with elevated PSA followed for up to 30 months, patients with low IsoPSA indices (for example, ≤6) demonstrated a very low subsequent risk of clinically significant prostate cancer (csPCa), with estimated risks of approximately 0.4%, 2.5%, and 6.3% at 12, 24, and 30 months, respectively, compared with roughly 5.9%, 31.7%, and 49.5% among those with high IsoPSA indices [261]. In this context, IsoPSA showed high sensitivity and specificity for csPCa and robust negative and positive predictive value at clinically relevant index thresholds, supporting its use to make more informed biopsy decisions in important clinical contexts (e.g., early detection, monitoring, active surveillance).

In addition, IsoPSA has been evaluated in combination with mpMRI and PI-RADS (Prostate Imaging Reporting and Data System) scoring to refine biopsy decision-making. In patients with elevated PSA who underwent MRI, an elevated IsoPSA ratio (for example, ≥6.0) and PI-RADS 4–5 lesions were both significant predictors of csPCa. Among men with negative or equivocal MRI findings (PI-RADS 1–3), a low IsoPSA ratio (≤6) was associated with a predicted csPCa probability of <5%, suggesting that integration of IsoPSA with mpMRI can improve risk stratification in equivocal imaging scenarios and may permit safe omission of biopsy in carefully selected patients [262]. Taken together, these data from validation, longitudinal, and mpMRI-integrated studies demonstrate statistically informative, reliable, and clinically meaningful IsoPSA performance for pre-biopsy risk stratification, with strong negative and positive predictive characteristics that directly support its role in guiding biopsy decisions [260,261,262].

## 8. Limitations and Challenges

The major limitation of the described approach is that although the SIA technique enables one to detect changes in protein structure and/or protein conformation and/or protein–partner (other protein, ligand, drug, etc.) interactions, it does not differentiate between these changes or identify them [89,94,269]. Additional techniques are required to resolve this issue. Furthermore, although a variety of factors affect protein partitioning in ATPSs (see Figure 4), the exact structural changes that escape detection by SIA are currently unknown. It is logical to presume, however, that any modifications leaving a protein’s interaction with the solvent completely unchanged will remain undetectable by this technique.

Since all proteins are characterized by unique structural traits and specific dynamic features that determine their partition behavior in ATPSs, no universal design strategy would guarantee an optimal selection of the ATPS composition for a given protein target in a single step. Instead, finding an optimal ATPS composition requires empirical, trial-and-error optimization [1,7,8,9,10]. The development of particular ATPS conditions for a new individual protein depends on the research purpose, e.g., analysis of its interactions with water, differentiation of mutants, or analysis of protein–ligand interactions. In any case, development starts with a small set of ATPSs formed by different pairs of polymers. Once ATPSs with a partition coefficient of 0.1 < K < 10 are found, the characterization of protein–water interactions can be performed. For analysis of different mutants or protein–ligand or receptor interactions, there are at least two states, e.g., free protein and protein–ligand complexes or wild-type protein and protein mutants. In this case, two or more different ATPSs providing sufficient difference between the pair of samples or states under consideration should be designed. For this purpose, there are generally two ways: (i) increase the concentrations of ATPS-forming polymers because this increases the difference between the compositions of phases and results in increasing the differences in the K-values for two samples, or (ii) changing the pH, buffer composition, or type and/or concentrations of salt additives [7].

For dynamic proteins with multiple conformational states (e.g., open/closed), the measured partition coefficient fundamentally reflects a population-weighted average across all accessible conformational states rather than resolving them into separate populations. This is because proteins typically interconvert between these accessible conformational states much faster than the time it takes to conduct a partitioning experiment. Therefore, the measured value is a macroscopic, time-averaged property of a query protein: an apparent partition coefficient representing a macroscopic, population-weighted average of all accessible states [7,94].

Another limitation regarding ATPS includes a lack of knowledge and understanding of which physicochemical properties of polymers define their ability to form aqueous two-phase systems. The effect of protein concentration on the partition coefficient measurements depends on the sensitivity of the test used for the measurement. The range of K-values for a protein may exceed several orders of magnitude. Under certain conditions, some proteins may precipitate at the interface. Although partitioning of a protein in ATPSs is governed by a combination of solvent interactions, structural features, and conformational dynamics, the measured K-value represents a global, macroscopic thermodynamic summation. Therefore, partitioning cannot be used to infer specific structural properties of a protein, indicating that SIA represents a kind of “fingerprint” approach rather than a tool for direct structural determination [89,94,95,145].

The limitation regarding clinical applications of SIA is the lack of information about low-abundance proteins that may likely serve as biomarkers. It seems that combining the SIA technique with Somalogic, Olink Proteomics, or Alamar screening technologies may advance the development of protein biomarkers. The throughput of the approach can be up to several hundred or more samples per day, depending on the liquid-handling equipment and concentration measurement technique employed.

According to the accumulated experimental data, the reproducibility error of the partition coefficient for a given protein is less than 5% across different laboratories. However, it can actually be reduced below 1% by using precise automated systems and strictly controlling environmental variables. The only problematic issue with the matrix of biofluid noticed so far is that urine samples from different patients could not be stored at −80 °C due to protein changes during storage.

Future applications include clinical tests for different types of oncology, development of a multicancer screening test [270], and tests for detection of neurological disorders. In addition, the general approach to detecting changes in protein structure and/or protein interactions with components of their environment under any physiological conditions may be developed by combining SIA technology with SomaScreen from Illumina/Somalogic (San Diego, Boulder, CO, USA), the Proximity Extension Assay (PEA) from Olink Proteomics AB/Thermo Fisher Scientific Inc. (Uppsala, Sweden), or Nulisa assays from Alamar Biosciences Inc. (Fremont, CA, USA).

## 9. Conclusions

In conclusion, this review highlights several critical paradigm shifts in molecular diagnostics and biophysical analysis.

First, solvent interaction analysis (SIA) stands out as a uniquely powerful methodology, offering unparalleled quantitative insights into protein–aqueous media interactions. Unlike conventional biophysical tools, SIA achieves an exceptional level of sensitivity, capturing subtle thermodynamic and hydration changes that were previously unmeasurable.

Second, the traditional reliance on monitoring the expression levels of protein biomarkers is proving inadequate for the molecular diagnostics of complex diseases. Quantitative abundance alone fails to account for the heterogeneous nature of these disorders, often leading to inconsistent diagnostic outcomes.

Finally, shifting the analytical focus to structural alterations in proteins represents a far more robust frontier. Detecting early changes in protein conformation, misfolding, or post-translational modifications can substantially improve the early detection of oncological and neurological disorders, ultimately paving the way for more timely and precise clinical interventions.

## Figures and Tables

**Table 1:** Partition coefficients for mutants of staphylococcal nuclease A [90] and mutants of bacteriophage T4 lysozyme [91,92]. ATPS #1: dextran–Ficoll-0.15 M NaCl-0.01 M NaPB; ATPS #2: dextran–Ficoll-0.15 M Na_2_SO_4_-0.01 M NaPB; ATPS #3: dextran–Ficoll-0.11 M NaPB.

Mutant	ATPS #1	ATPS #2	ATPS #3	Signature
Staphylococcal nuclease A				
WT	0.97 ± 0.01	0.42 ± 0.01	0.29 ± 0.02	0.00
I18L	1.14 ± 0.03	0.64 ± 0.04	0.46 ± 0.05	2.41
T33I	1.10 ± 0.02	0.68 ± 0.03	0.52 ± 0.01	2.86
L37I	1.33 ± 0.10	1.25 ± 0.12	0.63 ± 0.06	5.50
I72M	1.18 ± 0.07	0.73 ± 0.07	0.62 ± 0.02	3.48
T82V	0.96 ± 0.03	0.60 ± 0.03	0.45 ± 0.04	2.12
L108I	1.73 ± 0.24	0.86 ± 0.05	0.73 ± 0.14	4.48
L108V	1.43 ± 0.06	0.79 ± 0.01	0.67 ± 0.02	3.95
V114L	1.05 ± 0.06	0.25 ± 0.07	0.26 ± 0.03	2.36
Bacteriophage T4 lysozyme				
C54T/C97A	1.30 ± 0.02	0.90 ± 0.02	0.68 ± 0.02	0.00
G113E	1.19 ± 0.02	0.86 ± 0.02	0.76 ± 0.01	1.33
T115A	1.32 ± 0.02	0.88 ± 0.02	0.64 ± 0.01	0.47
T115A/S117A	1.30 ± 0.02	0.85 ± 0.01	0.65 ± 0.01	0.87
S117A/R119A	1.28 ± 0.02	0.91 ± 0.01	0.73 ± 0.01	1.13
R119A	1.35 ± 0.02	0.92 ± 0.02	0.73 ± 0.01	0.53

**Table 2:** Partition coefficients of human serum albumin (HSA) when exposed to various concentrations of different drugs, as indicated [115].

Drug	[Drug], μmol/L	K	Drug	[Drug], μmol/L	K
Oxacillin	0	0.203 ± 0.006	Verapamil	0	0.203 ± 0.006
	0.17	0.279 ± 0.008		0.05	0.236 ± 0.008
	0.23	0.303 ± 0.007		0.10	0.265 ± 0.008
	0.28	0.324 ± 0.009		0.15	0.299 ± 0.016
	0.34	0.353 ± 0.010		0.20	0.326 ± 0.012
Caffeine	0	0.203 ± 0.006		0.25	0.327 ± 0.002
	0.13	0.207 ± 0.003		0.31	0.334 ± 0.006
	0.26	0.211 ± 0.011	Cefmetazole	0	0.203 ± 0.006
	0.39	0.215 ± 0.005		0.07	0.208 ± 0.012
	0.51	0.219 ± 0.007		0.13	0.214 ± 0.006
	0.64	0.227 ± 0.008		0.20	0.217 ± 0.003
Warfarin	0	0.203 ± 0.006		0.26	0.221 ± 0.012
	0.08	0.227 ± 0.010		0.33	0.227 ± 0.015
	0.11	0.238 ± 0.010		0.40	0.232 ± 0.015
	0.15	0.238 ± 0.010	Theophylline	0	0.203 ± 0.006
	0.19	0.249 ± 0.010		0.14	0.253 ± 0.005
	0.23	0.255 ± 0.004		0.29	0.262 ± 0.006
Terbutalin	0	0.203 ± 0.006		0.42	0.261 ± 0.001
	0.09	0.224 ± 0.010		0.56	0.265 ± 0.005
	0.19	0.236 ± 0.008	Diltiazem HCl	0	0.203 ± 0.006
	0.28	0.244 ± 0.004		0.08	0.225 ± 0.004
	0.37	0.250 ± 0.007		0.17	0.236 ± 0.010
	0.46	0.255 ± 0.003		0.25	0.244 ± 0.011
	0.56	0.257 ± 0.008		0.33	0.249 ± 0.009
Atenolol	0	0.203 ± 0.006		0.41	0.253 ± 0.005
	0.10	0.230 ± 0.009		0.50	0.255 ± 0.001
	0.19	0.242 ± 0.014	Propranolol	0	0.203 ± 0.006
	0.29	0.247 ± 0.006		0.08	0.205 ± 0.005
	0.38	0.250 ± 0.007		0.17	0.210 ± 0.009
	0.48	0.252 ± 0.008		0.25	0.223 ± 0.014
	0.57	0.257 ± 0.005		0.34	0.237 ± 0.004
				0.42	0.251 ± 0.008
				0.51	0.270 ± 0.0015

## Data Availability

No new data were created or analyzed in this study. Data sharing is not applicable to this article.

## Abbreviations

The following abbreviations are used in this manuscript:

ATPS	Aqueous two-phase systems
AUC	Area under the receiver operating characteristic curve
BC	Biomolecular condensate
DNP	2,4-Dinitrophenol
CA-125	Cancer antigen 125
CDG	Congenital disorder of glycosylation
CDT	Carbohydrate-deficient transferrin
CR	Caloric restriction
CSF	Cerebrospinal fluid
ELISA	Enzyme-Linked Immunosorbent Assay
HBA	Hydrogen-bond acceptor
HBD	Hydrogen-bond donor
HG-PCa	High-grade prostate cancer
HPLC	High-performance liquid chromatography
HSA	Human serum albumin
IR	Infrared
iTRAQ	Isobaric tags for relative and absolute quantitation
LCST	Lower critical solution temperature
LG-PCa	Low-grade prostate cancer
LSER	Linear solvation energy relationship
LLPS	Liquid–liquid phase separation
MLO	Membrane-less organelle
mpMRI	Multiparametric magnetic resonance imaging
MS	Mass spectrometry
MSIA	Mass spectrometric immunoassay
OPA	o-Phthalaldehyde assay
PEG	Polyethylene glycol
PPG	Polypropylene glyco
PSA	Prostate-specific antigen
PTM	Post-translational modification
PVP	Polyvinylpyrrolidone
RF	Refeeding
SIA	Solvent interaction analysis
SWATH-MS	Sequential window acquisition of all theoretical mass spectra
TIPS	Temperature-induced phase separation
TLS	Tie-line length
TMAO	Trimethylamine N-oxide
UCST	Upper critical solution temperature
